# The 3rd Annual Ontario Thoracic Cancer Conference at Niagara-on-the-Lake

**Published:** 2008-06

**Authors:** M. Dahele, Y. Ung, A. Bezjak, R. Burkes, P. Ellis, A. Behzadi, H. Solow, S. Laffan, M. Korec

**Affiliations:** * Radiation Medicine Program, Princess Margaret Hospital, University Health Network, Toronto, ON; † University of Toronto, Toronto, ON; ‡ Department of Radiation Oncology, Odette Cancer Centre, Sunnybrook Health Sciences Centre, Toronto, ON; § Division of Medical Oncology, Mount Sinai Hospital and Princess Margaret Hospital, University Health Network, Toronto, ON; || Department of Medical Oncology, Juravinski Cancer Centre, Hamilton, ON; # McMaster University, Hamilton, ON; ** Department of Thoracic Surgery, Scarborough General Hospital, Scarborough, ON; †† Department of Medical Oncology and Hematology, Markham Stouffville Hospital, Markham, ON

**Keywords:** Thoracic cancer, lung cancer, Ontario, conference

## 1. INTRODUCTION

The 3rd Annual Ontario Thoracic Cancer Conference was held March 28–30, 2008, in Niagara-on-the-Lake, bringing together those in community and academic practice throughout the province who have an interest in thoracic oncology. More than 150 attendees participated this year, twice as many as participated at the inaugural meeting. For the first time, abstracts from trainees were submitted, and those abstracts are published here. The selected highlights that follow underscore the breadth of the work ongoing in Ontario to combat the challenge of lung and other thoracic cancers.

## 2. HIGHLIGHTS

### 2.1 Technological Innovations in Radiation Medicine

The first day’s keynote speaker, Dr. David Jaffray [Princess Margaret Hospital (pmh)], has been at the forefront of developments in cone-beam computed tomography (cbct) that have enabled a new era in image-guided radiation therapy (igrt) 1,2. Devices for cbct are now mounted on linear accelerators used for radiation therapy and provide three-dimensional volumetric imaging that permits greater accuracy and precision in identifying tumour position and in delivering treatment. Cone-beam computed tomography may also allow for an assessment of the treatment response of peripheral lung tumours, an important step in the development of adaptive radiation therapy that seeks to modify aspects of treatment according to response—for example, to increase the likelihood of response or to reduce normal-tissue toxicity 3.

Dr. Jaffray presented an overview of advances in radiation therapy for lung tumours. He discussed methods for overcoming the uncertainty in tumour location associated with respiratory motion during treatment, and he articulated the importance of taking this motion into account during the treatment planning process. Accounting for motion is now commonly achieved with the use of “four-dimensional” computed tomography (4dct) imaging, a process that sorts ct images into “bins” that correspond to various phases of the respiratory cycle. By combining images from the various phases (for example, inhale and exhale), tumour excursion can be approximated and included in the treatment volume. Currently, 4dct and cbct are both playing an important role in radioablative stereotactic lung radiation therapy, an emerging treatment option for certain lung tumours 4.

### 2.2 Evaluation of the Mediastinum in Lung Cancer

Accurate evaluation of nodal involvement in the mediastinum is a critical issue in thoracic oncology. Drs. Lisa Ehrlich (Sunnybrook Health Sciences Centre), Kam Soghrati [The Scarborough Hospital (tsh)], and Abdollah Behzadi (tsh) addressed the multimodal evaluation of the mediastinum. They covered the role of positron-emission tomography (pet)–ct imaging, the emerging option of endobronchial ultrasound (ebus) fine-needle aspiration cytology (fnac), and mediasti- 5. They noted that mediastinoscopy, like ebus, can be performed as a day-case procedure, and that it may also offer additional information to the surgeon about potential eligibility for trimodality therapy in cases of suspected mediastinal involvement. Mediastinos-copy has resolutely remained the “gold standard” despite a number of innovations in mediastinal assessment, but for how much longer that leading position will be maintained remains to be seen. Currently, several centres in Ontario are either offering or considering ebus. As with mediastinoscopy, ebus is subject to operator dependency, and it further requires close cooperation with the cytopathology team to realize its potential. When ebus is combined with endoscopic ultrasound (eus) fnac, a more complete mediastinal assessment is possible, and eus fnac can also assess some potential sites of metastatic disease as well 6. As with any sampling technique, the potential for false negative results with fnac should be borne in mind. Attendees heard how individual centers are taking steps to satisfy themselves that, in their hands, ebus can deliver results comparable to those with mediastinoscopy.

The indications for ^18^Fluorodeoxyglucose (fdg) pet–ct imaging are increasing 7. Currently, this imaging modality can be accessed in the province of Ontario through a combination of specified indications (pet registry studies), case-by-case application (through the pet Access Program), and clinical trials. Noninvasive pet is useful in evaluating the mediastinum, but there are potential pitfalls. Causes of false positive and false negative pet scans were reviewed, and attendees were reminded that, in certain circumstances—for example when the primary lung cancer has low fdg uptake—further investigation of a pet-negative mediastinum should be considered 8.As technologies develop and patient access to them increases, it seems likely that mediastinal assessment in thoracic cancers will be achieved through a combination of noninvasive and invasive approaches.

### 2.3 Setting Thoracic Surgical Standards in the Province of Ontario

In a session that generated much discussion, the challenge of redesigning provincial thoracic surgery services was tackled by Drs. Sudir Sundaresan (The Ottawa General Hospital) and A. John Dickie (Peterborough Regional Health Centre). They reviewed the setting of thoracic surgery standards by Cancer Care Ontario (cco) to distinguish level i from level ii surgical centers 9,10 and highlighted the many stakeholders in such a large-scale service redesign. The issue promises to be an emotive one, but there was broad support at the meeting for the setting of thoracic surgical standards so as to benefit patients and ultimately improve outcomes. The stakeholders range from local communities, some of which are geographically quite isolated and currently receive care from a community thoracic surgeon, to hospitals that gain both tangibly and intangibly from having a thoracic surgery service, to surgeons themselves.

The cco standards were developed in response to the question “What is the optimum organization for the delivery of cancer-related thoracic surgery in Ontario,” but as was pointed out, oncology is only one part of the thoracic surgeon’s practice. The potential effects of a service redesign of this kind on cancer *and* non-cancer services alike will require close attention. It was suggested during the discussion that, if significant redesign is undertaken, careful planning and a means of prospectively evaluating the effects should be built into the process.

### 2.4 Lung Cancer Patterns of Care

Given his longstanding interest in cancer service delivery, patterns of care, and outcomes, Dr. William Mackillop (Queen’s University) delivered a thought-provoking and somewhat sobering review of variations in the management and outcome of non-small-cell lung cancer (nsclc) in Ontario 11. Drawing on many years’ experience with the use of multisource population databases, he reviewed the development of such tools and described how they are being used. He presented data demonstrating that, although the outlook for selected patients may be improving, the overall outcome of all patients with nsclc has been little affected. Whether more recent data incorporating the rise in delivery of adjuvant chemotherapy for resected early-stage lung cancer and other therapeutics will change this finding remains to be seen 12. In pursuing maximization of outcomes for lung cancer patients on a province-wide scale, it seems that regional variation in therapy utilization and access to services needs to be better understood and explicitly considered in health care initiatives.

### 2.5 Contributions of the National Cancer Institute of Canada Clinical Trials Group

Dr. Frances Shepherd (pmh), chair of the Lung Site of the National Cancer Institute of Canada (ncic) Clinical Trials Group (ctg), described the significant impact made by that group on the international stage. Pivotal trials affecting lung cancer treatment were reviewed, including these studies:

- BR 6—In patients with limited-stage small-cell lung cancer receiving chemotherapy and radiation, early integration of thoracic radiation, as compared with late or consolidative radiation, resulted in better progression-free and overall survival 13.
- BR 10—In patients with completely resected, early-stage nsclc, adjuvant vinorelbine and cisplatin, as compared with observation, prolonged disease-free and overall survival 14.
- BR 21—In patients with advanced nsclc who progressed after 1 or 2 lines of prior chemotherapy, use of the epidermal growth factor receptor inhibitor erlotinib, as compared with placebo, resulted in better survival 15.

The ncic ctg continues to play a major role in national and international cooperative lung cancer studies. In doing so, it draws on an array of Canadian talent, including the expertise that exists within Ontario in such areas as molecular medicine, therapeutics, and quality-of-life and economic assessment.

Additional debate on the role of trimodality therapy in selected patients with stage iiia, non-bulky N2 disease {Drs. Gail Darling (Toronto General Hospital) and Gordon Okawara [Juravinski Cancer Centre]} (jcc) 16–19, discussion of the ethics of expensive cancer therapies [Dr. Scott Berry (Odette Cancer Centre)] 20, and exploration of the role of the advanced practice nurse in lung cancer [Ms. Lorraine Martelli–Reid (jcc)]^21^ made for a conference program that was both contemporary and practical. It was rounded out by the presentation of the top two scientific abstracts of the conference [Drs. Jason Correia (McMaster University) and Gerald Lim (PMH)], which were all subject to review by a panel of judges.

## 3. SUMMARY

It is hoped that, in future years, the conference will continue to grow and become an effective tool for fostering multidisciplinary, multicentre collaboration and for improving the outcome for lung cancer patients throughout the province of Ontario.

Many thanks to all those who made this meeting a success, including sponsors who provided unrestricted educational grants: Eli Lilly, Astra Zeneca, and Sanofi–Aventis.
