# Supplementary material for: The 3rd Annual Ontario Thoracic Cancer Conference at Niagara-on-the-Lake
Source: Curr Oncol. 2008 Jun;15(3):155–61. (PMC2442767)
Supplement: Related texts

# Abstract: Improving Outcomes for Early-Stage Non-Small-Cell Lung Cancer with Stereotactic Body Radiation Therapy

**Published:** 2008-06

**Authors:** M. Dahele, S. Pearson, T. Purdie, J.P. Bissonnette, A. Brade, J.B.C. Cho, A. Sun, A. Hope, K. Franks, D.A. Jaffray, A. Bezjak

**Affiliations:** Radiation Medicine Program, Princess Margaret Hospital, University Health Network, Toronto, ON; Department of Radiation Oncology, University of Toronto, Toronto, ON; St. James Institute of Oncology, The Leeds Teaching Hospitals NHS Trust, U.K

## Background and Objectives

About 20% of patients have early-stage non-small-cell lung cancer (nsclc). Medically inoperable patients may receive radical radiation therapy (rt). Conventional rt outcomes are far inferior to surgery. Delivered in only a few fractions, stereotactic body radiation therapy (sbrt) gives ablative doses of rt that are expected to provide high rates of local control.

## Methods

Patients with medically inoperable T1-2N0M0 nsclc are treated on Research Ethics Board–approved sbrt protocols. Patients lie in customized body moulds to minimize movement, and respiratory-synchronized computed tomography (ct) scans for treatment planning identify tumour position and motion. Multiple beams shape the radiation dose to the target. Image guidance by ct before and during treatment verifies patient and target position to 3 mm or less. Positron-emission tomography (pet) is used for staging and response assessment.

## Results

In 76 patients (median age: 73 years) with 82 lesions (median size: 2.3 cm), median follow-up is 8 months. In 49 patients (53 lesions), follow-up is 6 months or more (median: 13 months). In the latter group, 2 lesions (4%) have failed locally, 11 patients (22%) have metastases (median: 9 months), and 9 patients (18%) have died (median: 11 months). Overall, the 3-month radiologic response rate is 56% (39 of 69 lesions). The most common acute (within 3-months) toxicities are grades 1 or 2 fatigue (41%), cough (21%), skin erythema (14%), and chest wall pain (8%); late toxicities include grades 1 or 2 chest wall pain (23%), grades 1 through 3 rib fracture (14%), and grades 1 or 2 atelectasis (9%). Four grade 3 events have occurred (3 rib fractures, 1 pneumonitis).

## Conclusions

Treatment with sbrt is promising and well tolerated. Local control is high. Long-term data will define efficacy. Clinical indications are evolving; patients with oligometastatic nsclc and pulmonary metastases may benefit from sbrt.

# Abstract: Outcomes for Cervical Mediastinoscopy in a Community Teaching Hospital

**Published:** 2008-06

**Authors:** J. Lee, Y. Ung, A. Behzadi

**Affiliations:** Department of Surgery, The Scarborough Hospital; Department of Radiation Oncology, University of Toronto; and Odette Cancer Centre, Sunnybrook Health Sciences Centre, Toronto, ON

## Background

Cervical mediastinoscopy (cm) has been considered the “gold standard” for pathology assessment of mediastinal abnormalities. In view of the development of less invasive technologies for mediastinal evaluation and their dissemination to community hospital settings, we sought to determine the outcomes for cm in a community hospital by examining the complication and false negative rates of this procedure as a benchmark for future comparisons.

## Methods

We performed a retrospective chart review of 150 consecutive patients who underwent cm between January 2004 and December 2006 (inclusive). Demographics of the patients and the complications and results of the procedures performed were evaluated. In lung cancer patients who subsequently underwent thoracotomy, the false negative rate for cm was calculated.

## Results

Median age of the patients was 69 years, and 52% were men. Pathology included lung cancer (*n* = 105), metastatic disease (*n* = 6), lymphoma (*n* = 8), and benign diseases (*n* = 31). Postoperative complication rate was 2.7%. No postoperative deaths occurred. Of 56 patients with negative mediastinoscopy who underwent thoracotomy, 2 were later found to harbour positive N2 nodes in lymph node stations accessible by cm, for a false negative rate of 3.57%. Perioperative imaging of all false-positive cm cases were negative for evidence of N2 involvement.

## Conclusions

In this cohort of patients, cm was associated with no mortalities and low rates of postoperative complications and false-negative diagnostic imaging. The less-invasive endoscopic and radiologic technologies for mediastinal assessment should at least have the same false negative rates and lower complication rates if they are to replace cm in the community hospital setting.

# Abstract: Video-Assisted Thoracoscopic Surgery Lobectomy for Lung Cancer: Prospective Data on Initial Experience at a Canadian Community Hospital

**Published:** 2008-06

**Authors:** J.D. Correia, A.A. Walele, A.S. Ashrafi

**Affiliations:** Department of Surgery, McMaster University, Hamilton, ON, and Clinical Teaching Unit, St. Catharines General Hospital, Niagara Health System, ON

## Background and Objectives

Advantages of video-assisted thoracoscopic surgery (vats) lobectomy, as compared with lobectomy by conventional thoracotomy, include shorter hospital stay and reduced postoperative pain. We examined the preliminary experience with vats lobectomy in a community hospital.

## Methods

Data were prospectively collected on all patients that underwent vats lobectomy by a single surgeon at the St. Catharines General Hospital between August 2006 and November 2007. All patients were discharged on a standard narcotic protocol.

## Results

Lobectomy by vats was performed in 28 patients [43% men; median age: 67.5 years (range: 45–87 years)]. Preoperative percentage predicted forced expiratory volume in 1 s (fev1) was median 82.0% (range: 54%–120%) and percentage diffusion capacity (dlco) was median 73.5% (range: 44%–111%).

Pathology revealed that 82.1% (*n* = 23) of patients had primary non-small-cell lung cancer (nsclc) stage ia (39.1%), stage ib (43.5%), stage iia (4.3%), stage iib (8.7%), and stage iiib (4.3%). Other pathology included metastatic colorectal adenocarcinoma (*n* = 2), carcinoid (*n* = 1), hamartoma (*n* = 1), and pseudotumour (*n* = 1). All patients had complete resection with negative margins on pathology exam.

Two conversions to open thoracotomy for bleeding control occurred (conversion rate: 7.1%). Median postoperative chest-tube days were 4 (range: 2–13 days). Length of hospital stay was median 5.5 days (range: 3–44 days). No deaths were reported at 30-days of follow-up.

## Discussion and Conclusions

Lobectomy by vats for lung cancer can be performed safely in a community hospital with acceptable morbidity and mortality. Lobectomy by vats is a feasible alternative to conventional thoracotomy in the management of lung cancer.

# Abstract: The Long Journey of Lung Cancer Patients to Treatment

**Published:** 2008-06

**Authors:** A. Sturdza, D. Payne, A. Sun, J.B.C. Cho, G. Kane, D. Hodgson, J. Waldron, J. Catapano, F.A. Shepherd, S. Keshavjee, A. Bezjak

**Affiliations:** Princess Margaret Hospital, University Health Network; and University of Toronto, Toronto, ON

## Background and Objectives

Treatment recommendations for lung cancer patients require accurate staging and multidisciplinary assessment. Our aim was to assess the delays to treatment decision-making in patients referred to our institution for consideration of radical radiation.

## Methods

Retrospective chart review of patients referred to the Princess Margaret Hospital Lung Radiation Oncology Group with lung cancer from January 1 to September 30, 2004, documented dates and findings related to the critical steps leading to treatment decision.

## Results

Among 284 patients referred with presumed non-metastatic disease, the following intervals were documented: chest X-ray (cxr) to initial computed tomography (ct) imaging, median 28 days; and ct imaging to tissue biopsy, median 22 days. In 174 patients (61%), further imaging in our institution to update old chest ct images or because of development of symptoms was needed for completion of metastatic workup. Repeat ct chest showed progression of the primary or lymph nodes in 85 patients (56%; median 70 days from the initial ct), upstaging to incurable disease in 22 patients (13%), and no change in 32 patients (21%). A stage change occurred in 33% of patients after completion of their workup, with most being upstaged to stage iv. After completion of assessment, only 118 patients (41%) were treated radically, at a median of 3.2 months from initial cxr.

## Discussion and Conclusions

Our review documented extensive delays at multiple steps, adding to more than 3 months from initial cxr to the start of radiotherapy. These time delays were associated with progression of cancer. Expediting multidisciplinary assessment of patients with suspected lung cancer could result in improved outcomes.

# Abstract: Synergistic Antitumour Effects of Regulatory T-Cell Blockade Combined with Pemetrexed in Murine Malignant Mesothelioma

**Published:** 2008-06

**Authors:** M. Anraku, Z. Yun, S. Keshavjee, L. Zhang, M.R. Johnston, M. de Perrot

**Affiliations:** Latner Thoracic Surgery Research Laboratories, Toronto General Research Institute, University Health Network, University of Toronto, ON

## Background/objectives

CD4^+^CD25^+^ regulatory T cells (“Tregs”) can promote the growth of some tumours. We previously showed that the presence of Tregs was associated with poor survival of patients with malignant pleural mesothelioma). The objective of the present study was to determine whether Treg blockade might enhance survival when combined with pemetrexed in established tumours in our intrathoracic murine model of malignant mesothelioma.

## Materials and Methods

Murine malignant mesothelioma cells (AC29) were injected into the right pleural cavity of cba mice for tumour development. Four days after the injection, tumour-bearing mice were treated with pemetrexed with or without Treg blockade.

## Results

The synergistic antitumour effect of Treg blockade and pemetrexed on prolonged survival was associated with increased interleukin 2 and suppressed transforming growth factor β1 at the local tumour site. Significantly decreased %Treg and increased %CD8^+^ cytotoxic T cells were observed in mice treated with the combined protocol, but not in mice treated with pemetrexed alone. The survival benefit was abrogated if anti-CD8 monoclonal antibody was simultaneously administered.

## Conclusions

Our findings suggest that Treg blockade combined with pemetrexed can suppress mesothelioma growth in established tumour *in vivo* by inducing greater antitumour immune reaction. This study also validates a new intrathoracic tumour model of pleural effusion to explore the role of antitumour immunity in murine malignant mesothelioma.

# Abstract: Prognostic Factors in Solitary Resected Brain Metastasis from Non-Small-Cell Lung Cancer

**Published:** 2008-06

**Authors:** A. Louie, G. Rodrigues, F. Whiston, L. Stitt

**Affiliations:** Department of Oncology, Division of Radiation Oncology, London Regional Cancer Program; and Schulich School of Medicine and Dentistry at the University of Western Ontario, London, ON

## Background and Objectives

Reports in the medical literature have described cases of extended survival of patients with stage iv non-small-cell lung cancer (nsclc) with solitary metastatic disease that have received aggressive management both to the brain metastasis as well as to the local or regional disease. The objective of the present research was therefore to analyze prognostic factors that predict for outcome in this unique patient population.

## Methods

We performed a single-institution retrospective chart review of 35 nsclc patients with solitary brain metastasis treated with craniotomy and whole-brain radiotherapy. Of these patients, 8 (22.9%) underwent chest surgery, 24 (68.6%) received chemotherapy, and 14 (40.0%) received thoracic radiation; 13 were otherwise stage i/ii (37.1%), and 20 were stage iii (57.1%). Mean age at diagnosis was 58.5 years. Brain metastasis <3 cm was present in 18 (56.25%) and >3 cm in 14 (43.75%).

## Results

Median survival was 7.8 months. At last follow-up, 3 patients (8.6%) were alive and well, 6 (17.1%) were alive with disease, 24 (68.6%) had died of their disease, and 2 (5.7%) had died of other causes. Univariate analysis demonstrated that lung surgery (*p* = 0.0033), primary lung treatment more than 8 weeks after brain surgery (*p* = 0.0128), and stage i/ii (*p* = 0.0467) were predictive of overall survival.

## Discussion and Conclusions

Survival remains poor for stage iv nsclc patients with a synchronous solitary brain metastasis. However, patients with thoracic disease amenable to local resection should be considered for such therapy, because it may hold a survival advantage for them as compared with patients with more locally-advanced disease.

# Abstract: Hyperpolarized ^3^He Magnetic Resonance Imaging of Radiation Pneumonitis: Initial Findings at 3.0 Tesla

**Published:** 2008-06

**Authors:** L. Mathew, G. Rodrigues, G. Parraga

**Affiliations:** Imaging Research Laboratories, Robarts Research Institute; Department of Medical Biophysics; Department of Oncology, The University of Western Ontario; Lawson Health Research Institute; London Regional Cancer Program, London Health Sciences Centre, London, ON

## Background and Objectives

Hyperpolarized ^3^He magnetic resonance imaging (hp 3He mri) is a method of functional lung imaging allowing for visualization of ventilation patterns and defects at a high resolution. In this pilot study, we assessed potential ^3^He mri phenotypes of radiation pneumonitis in lung cancer patients post-radiation.

## Methods

Subjects were recruited from the London Regional Cancer Program based on post-radiation clinical or radiologic evidence of pneumonitis and spirometric measurement of forced expiratory volume in 1 s. We scanned 4 subjects using hp ^3^He mri imaging at 3.0 Tesla. Imaging occurred during a 16-s breath-hold of hp ^3^He, using a spin-exchange optical pumping system. The static ventilation images thus acquired enabled analysis of ventilation patterns. A breath-hold-matched proton scan was also acquired to allow for visualization of the thoracic cavity for anatomic comparison.

## Results

Preliminary results in 4 subjects suggest that numerous changes occur in the ventilation patterns of the lungs of subjects with pneumonitis following radiation therapy. Ventilation defects were found distal to, and in some cases coinciding with, treatment site and radiation pneumonitis location in subjects with radiation pneumonitis resulting from lung cancer treatment. Quantitative analysis of ventilation defect volume and direct comparison to X-ray computed tomography results provide an explanation of the causes of ventilation defects.

## Conclusions and Discussion

^3^He mri is a valuable tool for the visualization of regional damage resulting from pneumonitis post-radiation. Analysis of radiation treatment parameters in conjunction with ^3^He mri may allow for improved understanding of lung response to radiation.

# Abstract: Effects of Genistein Following Fractionated Lung Irradiation

**Published:** 2008-06

**Authors:** A.E. Para, V.L. Calveley, I.W.T. Yeung, J. Van Dyk, R.P. Hill

**Affiliations:** Departments of Medical Biophysics and of Radiation Oncology, University of Toronto; Research Division, Ontario Cancer Institute/ Princess Margaret Hospital, and Radiation Medicine Program, Princess Margaret Hospital, University Health Network, Toronto, ON; and Radiation Treatment Program, London Regional Cancer Program, London Health Sciences Centre; and Department of Oncology and Medical Biophysics, University of Western Ontario, London, ON

## Introduction

We hypothesize that inflammatory cytokines and the subsequent production of reactive oxygen species play a key role in radiation-induced lung injury. Previously, we showed that genistein is effective in preventing damage following single doses of radiation. The purpose of the present study was to investigate protection by genistein with fractionated doses of radiation.

## Methods

C3H/HEJ mice were irradiated with a dose of 9 fractions of 3.1 Gy over 30 days (approximately 10 Gy single dose) after having been maintained on a genistein diet (equivalent to approximately 10 mg/kg) just before the first fraction. Damage was assessed over 28 weeks in cells obtained from the lungs by a cytokinesis block micronucleus (mn) assay and by monitoring changes in breathing rate and histology. Tumour protection was also assessed by colony assay to determine cell survival following *in situ* irradiation.

## Results

Genistein was found to cause about a 50% reduction in the mn damage observed during the fractionated treatment and to continue to reduce mn formation at later times to background levels by 16 weeks. Genistein also reduced collagen deposition by 30%. A slight protection against increases in breathing rate were noted during weeks 11–18, but not at later times. This protection did not increase overall survival. Using the KHT fibrosarcoma model, we demonstrated no tumour protection by genistein following single and fractionated radiation doses.

## Conclusions

Our results suggest that genistein can protect lung cells against damage caused by irradiation, but that such protection does not extend to tumour cells growing in the lung.

# Abstract: An Institutional Review of the Current Medical Chart Documentation Practices for Tobacco Use in Radiation Oncology Patients with Lung or Head-and-Neck Cancer

**Published:** 2008-06

**Authors:** L. Pan, J. Cohen, G. Okawara, P. Nair

**Affiliations:** Juravinski Cancer Centre, McMaster University, and Firestone Institute for Respiratory Health, St. Joseph’s Hospital, Hamilton, ON; and University of Toronto and Ontario Tobacco Research Unit, Toronto, ON

## Background

Smoking during radiation therapy increases the risk of treatment failure and disease recurrence. In fact, complete response rates are significantly lower in patients who continue to smoke during treatment. Despite this, smoking cessation is seldom adequately addressed in tertiary cancer centres.

## Objectives

To retrospectively review medical chart documentation practices of radiation oncologists for tobacco use in patients with lung or head-and-neck cancer seen in consultation at a tertiary care cancer centre.

## Methods

The 134 charts examined for current radiation oncology patients consisted of 67 consecutive charts in each of two tumour sites. Charts were reviewed to determine the information documented by physicians on the patients’ smoking status and any cessation counselling offered.

## Results

Overall, information pertaining to smoking status was documented in 104 of 134 (77.6%) cases. Analysis by tumour type showed 79.1% documentation of smoking status in lung cancer and 76.1% in head-and-neck cancer. Smoking status information meeting a predetermined threshold level of adequacy required in patient management was found in 67% of the charts overall. History regarding second-hand smoke exposure was recorded in only 2% of charts. Lack of smoking status documentation occurred more frequently in patients who had been previously seen by medical or surgical oncology (30%) and in cases of treatment with palliative intent (30%). Documented smoking cessation counselling by the oncologist occurred in 1 of 134 cases.

## Conclusions and Discussion

With only 67% of patient charts in this population containing adequate smoking status information, there appears to be significant room for improvement. Adequate recording of tobacco use status is instrumental as the first step in any effort to prompt cessation counselling in subsequent follow-up visits. Our study emphasizes the need for more attention to smoking cessation efforts in oncology patients.

# Abstract: Smoking Cessation in Radiation Oncology as the Missing Drug in Clinical Trials—Identifying Patterns of Tobacco Use and Barriers to Cessation in Oncology Patients

**Published:** 2008-06

**Authors:** L. Pan, J. Cohen, P. Nair, G. Okawara

**Affiliations:** Juravinski Cancer Centre, McMaster University, and Firestone Institute for Respiratory Health, St. Joseph’s Hospital, Hamilton, ON; and University of Toronto and Ontario Tobacco Research Unit, Toronto, ON

## Background

Abstinence from tobacco use, especially during treatment, may significantly improve response rates to radiation therapy. The magnitude of this improvement may be greater than that seen with drugs such as radiosensitizers or chemotherapy. Nevertheless, some patients continue to smoke during radiation therapy.

## Objectives

To perform a qualitative study to examine the tobacco use behaviours and barriers to smoking cessation of patients diagnosed with lung or head-and-neck cancer.

## Methods

The study accrued 18 patients with a diagnosis of either lung or head-and-neck cancer who were on active radiation therapy at a tertiary care cancer centre. Detailed self-administered questionnaires or structured interviews (or both) were used to gather data regarding smoking behaviours, barriers to cessation, and attitudes toward tobacco use cessation. Input was also obtained from key health care providers.

## Results

Smoking cessation represented a difficult achievement for most patients, requiring multiple attempts. The most important reason for quitting cited by former smokers was the patient’s cancer diagnosis. The most significant patient-perceived barriers to smoking cessation included a belief of being addicted to cigarettes, using cigarettes to “calm nerves,” cravings for a cigarette, and fear of losing the enjoyment or pleasure derived from smoking. Overall, patients’ perception that smoking is bad for one’s health increased after their diagnosis of malignancy. For most patients, self-reported overall quality of life improved or remained the same after quitting. Practitioner-based barriers to tobacco-use cessation counselling primarily included a lack of time in clinical consultation visits.

## Conclusions and Discussion

Knowledge gained from this study is instrumental for ongoing program development and may contribute to investment in resources for tobacco use cessation efforts in the oncology population.

# Abstract: Toxicity of an Accelerated Hypofractionated Radiotherapy Regimen for Early-Stage Non-Small-Cell Lung Cancer

**Published:** 2008-06

**Authors:** H. Soliman, P. Cheung, I. Poon, J. Balogh, L. Barbera, J. Spayne, C. Danjoux, M. Dahele, Y. Ung

**Affiliations:** Odette Cancer Centre, Sunnybrook Health Sciences Centre, Department of Radiation Oncology, University of Toronto, Toronto, ON

## Background and Objectives

To retrospectively review the toxicity profile of an accelerated hypofractionated radiotherapy alone for early-stage non-small-cell lung cancer (nsclc) in patients treated at a single institution.

## Methods

A policy at our institution of treating early-stage nsclc without nodal involvement with an accelerated hypo-fractionated regimen of 4 Gy daily was adopted in 1996. We report here the acute and late side effects associated with that treatment.

## Results

A total of 124 tumours were treated in 118 patients (56 men, 62 women). The median follow-up from end of treatment to last follow-up or death was 23.2 months. The median age at diagnosis was 74.7 years. Almost all patients (96.6%) were not candidates for surgery because of medical comorbidities or old age. All patients were node-negative, with 75 T1 tumours, 40 T2 tumours, and 9 T3/4 tumours. Most patients were treated with 48 Gy in 12 fractions (59.7%); the remainder were treated to 52 Gy in 13 fractions. The most common acute side effect was dermatitis (19.4%), followed by cough (13.7%), dyspnea (6.5%), esophagitis (4.8%), and chest pain (2.4%). Pneumonitis requiring steroids was reported in 5 patients, with 1 patient dying just 6 weeks after administration of steroids. Four rib fractures in the treatment field were reported. Acute side effects were well tolerated with no toxicity-related delay in completion of treatment.

## Conclusions

Accelerated hypofractionated radiotherapy with this dose fractionation is well tolerated in patients with early stage nsclc who are medically inoperable or refuse surgery.

# Abstract: Evaluation of Lung Tumour Regression with Four-Dimensional Cone Beam Computed Tomography for Adaptive Radiotherapy

**Published:** 2008-06

**Authors:** G. Lim, A. Bezjak, J. Higgins, D. Moseley, D. Hope, A. Sun, J. Cho, A. Brade, L. Le, J.P. Bissonnette

**Affiliations:** Princess Margaret Hospital, University Health Network, and University of Toronto, Toronto, ON

## Background and Objectives

With the advent of on-treatment image guidance technology such as cone-beam computed tomography (ct) scanning, radiation oncologists are now better able to define the changing tumour target over the 6- to 7-week course of radical lung cancer treatment. The objective of our project was to use respiratory-correlated cone-beam ct (rccbt) imaging to study the volumetric, positional, and geometric changes that occur throughout the course of radical radiotherapy in non-small-cell lung cancer (nsclc) to guide future research into adaptive radiotherapy for lung cancer patients.

## Methods

Patients with pathology-confirmed nsclc receiving radical radiotherapy to a dose of 45 Gy or higher with concurrent chemotherapy were included in the study. Serial cone-beam ct images were used to identify and contour the tumour in the exhale phase of respiration on successive weeks after the start of radiotherapy.

## Results

We evaluated 13 consecutive lung patients for tumour regression. In 8 patients, rccbct images were of sufficient quality to assess tumour regression. Tumour regression was identifiable in 5 of the 8 patients after 15 fractions and in 7 of the 8 patients after 20 fractions. We observed 19%–54% tumour shrinkage after 20 fractions and 14%–71% tumour shrinkage at the end of treatment.

## Discussion and Conclusions

Current rccbct image quality allows for assessment of tumours located more peripherally. In such cases, tumour regression is documented in most patients. The feasibility of adaptive radio-therapy in radical lung cancer treatment should be further investigated.
